# Prevalence and factors associated with *trichomonas vaginalis* infection among female sex workers in Togo, 2017

**DOI:** 10.1186/s12879-021-06432-w

**Published:** 2021-08-09

**Authors:** Martin Kouame TCHANKONI, Alexandra Marie Bitty-Anderson, Arnold Junior SADIO, Fifonsi Adjidossi GBEASOR-KOMLANVI, Valentine Marie FERRÉ, Wendpouiré Ida Carine ZIDA-COMPAORE, Ameyo Monique DORKENOO, Bayaki SAKA, Anoumou Claver DAGNRA, Charlotte CHARPENTIER, Didier Koumavi EKOUEVI

**Affiliations:** 1 Centre Africain de Recherche en Epidémiologie et en Santé Publique (CARESP), Lomé, Togo; 2 Centre de recherche PACCI-Site ANRS Côte d’Ivoire, Abidjan, Côte d’Ivoire; 3 Département de Santé Publique, Université de Lomé, Faculté des Sciences de la Santé, Lomé, Togo; 4 Université de Paris, INSERM UMR 1137 IAME, F-75018 Paris, France; 5 Laboratoire de Virologie, AP-HP, Hôpital Bichat-Claude Bernard, F-75018 Paris, France; 6 Département des Sciences Fondamentales, Université de Lomé, Faculté des Sciences de la Santé, Lomé, Togo; 7 CHU Sylvanus Olympio, Service de Dermatologie et Vénérologie, Université de Lomé, Lomé, Togo; 8 Programme national de lutte contre le sida, les hépatites virales, et les infections sexuellement transmissibles, Lomé, Togo; 9 ISPED, Université de Bordeaux & Centre INSERM U1219 - Bordeaux Population Health, Bordeaux, France; 10 Université de Lomé, Laboratoire de Biologie Moléculaire et d’Immunologie, Lomé, Togo

**Keywords:** *Trichomonas vaginalis*, Female sex workers, HIV, West Africa

## Abstract

**Background:**

The aim of this study was to estimate the prevalence and factors associated with *Trichomonas vaginalis* (*T. vaginalis*) among female sex workers (FSW) in Togo in 2017. A cross-sectional bio-behavioral study was conducted from August to October 2017 using a respondent-driven sampling method in four cities in Togo.

**Method:**

A standardized questionnaire was used to record socio-demographic data and sexual behavior patterns. *T. vaginalis* detection by molecular biology tests was performed using Allplex STI Essential Assay which detect also 6 others micro-organisms. A blood sample was drawn and serological test using SD Bioline Duo VIH/Syphilis rapid test was performed for Human immunodeficiency virus (HIV) and syphilis testing.

**Results:**

A total of 310 FSW with median age 25 years, interquartile range (IQR) [21–32 years] were included. The prevalence of *T. vaginalis* was 6.5% (95%CI = [4.1–9.9]) and, overall, prevalence of other STI ranged from 4.2% (95%CI = [2.3–7.2]) for *N. gonorrhoeae* to 10.6% (95% CI = [7.5–14.7]) for HIV. Binary logistic regression was conducted to assess factors associated with *T. vaginalis* infection. Living in Lomé (aOR = 3.19; 95%CI = [1.11–11.49]), having had sexual intercourse before the age of 18 (aOR = 5.72; 95%CI = [1.13–10.89]), and being infected with *C. trachomatis* (aOR = 3.74; 95%CI = [2.95–12.25]) were factors associated with *T. vaginalis* among FSW.

**Conclusion:**

The prevalence of *T. vaginalis* infection using molecular test was low among FSW in Togo. Extensive studies are needed to confirm and to better understand the epidemiology of *T. vaginalis* among this population and in other populations in Togo.

## Background

*Trichomonas vaginalis (T. vaginalis)* is the most common, curable parasitic sexually transmitted infection (STI) worldwide affecting both men and women [1]. In 2012, 143 million cases of *T. vaginalis* had been diagnosed in women aged 15–49 years worldwide, including 17.5 million in Africa [2]. In 2016, Bayesian meta-analysis was used to generate estimates of the prevalence of STI. In women, prevalence estimates for *T. vaginalis* was 5.3%, for *Chlamydia Trachomatis (C. trachomatis)* 3.8%, for *Neisseria gonorrhoeae (N. gonorrhoeae)* 0.9%, and for syphilis 0.5%. In men, prevalence estimates for *T. vaginalis* was 0.6% which was very low compared to *C. trachomatis* (2.7%) and *N. gonorrhoeae* (0.7%) [3]. *T. vaginalis* vaginal infection in the African region are estimated at 42.8 million, and in the same region, this infection is ten times more common in women than in men [4]. In Africa, data on clinical presentation and microbiological factors associated with *T. vaginalis* infection are limited [5]. In contrast, multiple studies on *T. vaginalis* have been performed in other regions of the world [6–9].

As with most STI, *T. vaginalis* infection is largely associated with an increased risk in Human immunodeficiency virus (HIV) acquisition [10–13]. In a meta-analysis of 11 studies, *T. vaginalis* infection was a risk factor for HIV (95% CI 1.3 to 1.7; *p* < 0.001) [14]. Data on *T. vaginalis* are mainly described in pregnant women [15, 16] and few data are available on key populations as populations with high risk of HIV infection. In Lagos (Nigeria), the prevalence of *T. vaginalis* infection among HIV-positive and HIV-negative pregnant women was 10.0 and 8.1%, respectively (*p* = 0.559) [17]. In Togo, HIV prevalence is five times higher (10.6%) among female sex workers (FSW) [18]) than in the general population (2.3% [19]).

To our knowledge, no study on *T. vaginalis* infection using molecular technique has been conducted in Togo, especially among FSW. The aim of this study was to estimate the prevalence and factors associated with *T. vaginalis* among FSW in Togo.

## Methods

### Study design and recruitment

Between August and October 2017, a cross-sectional study was conducted among FSWs in Togo. Covering 57,000 km^2^, Togo is a country located in West Africa with 7.6 million inhabitant in 2018. The HIV prevalence in the general population was estimated at 2.3% in 2018 [19].

Participants were included through the respondent-driven sampling method [20, 21]. FSWs were defined as women who had sex for money as compensation in the previous 12 months. Additional inclusion criteria were being ≥18 years old, living in Togo for more than 3 months, and giving written informed consent [22, 23].

### Study procedures and detection of *T. vaginalis* and other STI

A standardized questionnaire adapted from a Family Health International (FHI) 360 validated guide for bio-behavioral surveys was administered during a face-to face interview to collect information regarding socio-demographic characteristics, risky sexual behaviors.

Cervical swabs collection and venous blood sample as well as biological analyses were described in detail elsewhere [18].

### Statistical analysis

Descriptive analyses were performed and results were presented with frequency tabulations and percentages. Prevalence were estimated with their 95% confidence interval (95%CI). Chi-square or Fisher’s exact tests were used to compare categorical variables. In multivariable analysis, logistic regression was conducted to identify factors associated with *T. vaginalis* infection.

Associations in the regression model were expressed as adjusted odds ratio (AOR) using all variables that had *p* < 0.20 in the univariable regression. Predictor variables were selected as those found to be relevant according to the literature review. All computations were conducted using R© version 3.4.3 software and the level of significance was set at 5%.

## Results

### Socio-demographic and clinical characteristics

A total of 310 FSW with median age of 25 years, interquartile range (IQR) [21–32 years] participated in the study. Almost half (46.2%) of them were over 25 years old while around one third (30.3%) were between 21 and 25 years old. More than seven out of ten FSW (76.1%) reported living with their partner and the majority had at least a secondary school level (74.5%). Almost 80% of FSW had their first sexual intercourse before the age of 18. Sociodemographic characteristics according to geographic area are summarized in Table 1 and none of the differences observed were statistically significant.

**Table 1:** Sociodemographic characteristics of FSW in Togo, *N* = 310 (2017)

	Other cities ***N*** = 132	Lomé (capital city) ***N*** = 178	Total ***N*** = 310	P*
**Age (years), n (%)**				0.053
[18–20]	38 (28.8)	35 (19.7)	73 (23.5)	
[21–25]	43 (32.6)	51 (28.7)	94 (30.3)	
> 25	51 (38.6)	92 (51.6)	143 (46.2)	
**Living with a partner, n (%)**				0.879
Yes	100 (75.8)	136 (76.4)	236 (76.1)	
No	32 (24.2)	42 (23.6)	74 (23.9)	
**Education level, n (%)**				0.490
Primary school or below	38 (28.8)	41 (23.0)	79 (25.5)	
Secondary school	83 (62.9)	119 (66.9)	202 (65.1)	
At least high school	11 (8.3)	18 (10.1)	29 (9.4)	
**Age at first intercourse (years), n (%)**				0.126
≤ 15	41 (31.1)	47 (26.4)	88 (28.4)	
]15–18]	72 (54.5)	89 (50.0)	161 (51.9)	
> 18	19 (14.4)	42 (23.6)	61 (19.7)	

*Chi square test

### Prevalence of *T. vaginalis* infection and others STI

Table 2 summarizes the prevalence of sexually transmitted infections among FSW in Togo in 2017. The prevalence of *T. vaginalis* was 6.5% (95%CI = [4.1–9.9]) and, overall, prevalence of other STI ranged from 4.2% (95%CI = [2.3–7.2]) for *N. gonorrhoeae* to 10.6% (95%CI = [7.5–14.7]) for HIV.

**Table 2:** Prevalence of *Trichomonas vaginalis and other STI* among female sex workers in Togo (2017)

	N	n	Prevalence (%)	95CI%
**Trichomonas vaginalis**	310	20	6.5	[4.1–9.9]
**HIV**	310	33	10.6	[7.5–14.7]
**Mycoplasma genitalium**	310	17	5.5	[3.3–8.8]
**Chlamydia trachomatis**	310	19	6.1	[3.8–9.6]
***Neisseria gonorrhoeae***	310	13	4.2	[2.3–7.2]

*95%CI* 95% Confidence interval; *HIV* Human immunodeficiency virus

### Factors associated with *T. vaginalis* infection among FSW

After multivariable adjustment, three factors had a statistically significant positive association with *T. vaginalis* infection among FSW. FSW living in Lomé (aOR = 3.19; 95%CI = [1.11–11.49]), those having had sexual intercourse before the age of 18 (aOR = 5.72; 95%CI = [1.13–10.89]), and those being infected with *C. trachomatis* (aOR = 3.74; 95%CI = [2.95–12.25]) were more likely to have a *T. vaginalis* infection (Table 3).

**Table 3:** Factors associated with *Trichomonas vaginalis* among female sex workers in Togo, 2017 (*N* = 310)

	Univariable			Multivariable		
	OR	95%CI	***p***-value	aOR	95%CI	***p***-value
**Age (years)**						
≤ 24	–					–
> 24	0.58	[0.22–1.45]	0.249			–
**Geographic area**						
Others	–					–
Lomé	3.16	[1.13–11.24]	**0.044**	3.19	[1.11–11.49]	**0.045**
**Living with a partner**						
Yes	–					–
No	0.54	[0.12–1.68]	0.343			–
**Education level**						
Primary school or below	–					–
Secondary school	1.10	[0.41–3.51]	0.857			–
High school	0.53	[0.03–3.47]	0.568			–
**Age at first sex (years)**						
> 18	–					–
≤ 18	4.96	[1.01–8.90]	**0.122**	5.72	[1.13–10.89]	**0.029**
**HIV status**						
Negative	–					–
Positive	0.42	[0.02–2.15]	0.411			–
***Mycoplasma genitalium***						
Negative	–					–
Positive	2.04	[0.31–8.00]	0.368			–
***Neisseria gonorrhoeae***						
Negative	–					–
Positive	1.22	[0.07–6.71]	0.853			–
***Chlamydia trachomatis***						
Negative	–					–
Positive	4.58	[1.20–14.44]	**0.014**	3.74	[2.95–12.25]	**0.038**

*OR* Odds Ratio; *aOR*adjusted Odds Ratio.

*95%CI* 95% Confidence interval.

## Discussion

This study provided an update on the epidemiology of *T. vaginalis* infection and revealed a prevalence of 6.5% among FSW in Togo. The overall prevalence of other STI were 10.6, 5.5, 6.1 and 4.2% for HIV, *M. genitalium, C. Trachomatis* and *N. gonorrhoeae* infections respectively. Among FSW population, risk factors associated with *T. vaginalis* infection were the geographic area (capital city, Lomé), lower age at first intercourse and infection with *C. Trachomatis*.

The prevalence of *T. vaginalis* infection among FSW reported in this study is approximately similar to those reported across other countries, as reported by other studies. In Rwanda, a descriptive cross-sectional study conducted in 2015 among 1168 FSW reported a prevalence of 11.9% [24]. In a 2-year longitudinal study conducted among 350 Kenyan FSW, baseline prevalence of *T. vaginalis* was 9.2% [25]. In an another prospective cohort study among 352 South African youths including lesbian, gay, bisexual, transgender, and queer (LGBTQ), an overall prevalence of 4.8% has been reported (8.1% among female and 0.7% among male participants) [26]. In a prospective, interventional cohort study of FSW aged 18 to 25 years in Ouagadougou among 321 HIV-uninfected FSW the prevalence of *T. vaginalis* was 3% [27]. The prevalence of *T. vaginalis* however was reportedly lower in the general population of women in a prospective study among 302 pregnant women conducted in 2011 in Togo which found a prevalence of *T. vaginalis* of 3.7% [28]. There is a need for additional effort towards the prevention of STI among FSW, which is an occupational risk in this line of work that could be prevented by the correct and consistent use of condoms. Among FSW, the correct and consistent use of condoms is still a challenge that requires more attention [29, 30].

No other STI, except from *C. trachomatis* was associated with *T. vaginalis* infection. There was no association between *T. vaginalis* and HIV infection which is contrary to results from previous studies. In South Africa, an association was found between *T. vaginalis* and HIV positive infection (OR = 1.6; *p* = 0.041) [5]. Another study conducted in Ouagadougou found an association between HIV infection and *T. vaginalis* (aOR = 9.63; 95% CI: [2.93 to 31.59]) [27]. A meta- analysis of *T. vaginalis* and HIV infection in sub-Saharan Africa found that individuals infected with *T. vaginalis* were 1.5 times more likely to acquire HIV compared to individuals not infected with *T. vaginalis* [14]. However, as corroborated by other studies, *C. trachomatis* infection (adjusted Prevalence Ratio (aPR) = 8.53; 95%CI = [3.35–21.71]) was identified as a risk factor of *T. vaginalis* infection in Kenya [25]. In the same study, a significant association was reported between positive HIV status and *T. vaginalis* infection (aPR = 3.01; 95% CI = [1.45–6.24]). In another study conducted in China, *C. trachomatis* was associated to *T. vaginalis* infection (aOR = 2.4 [95% CI: 1.37–4.14]) [31]. The lack of the association between HIV and *T vaginalis* in our study could be explained by the large access to antiretroviral treatment for the FSW when identified HIV positive, however this information is not collected. In addition, the lack of association between HIV, *M. genitalium, C. trachomatis* and *N. gonorrhoeae* infections could be explained by several factors including the limited sample size of the study population. Studies could further explore the relationship between other STI infection and *T. vaginalis* in high-risk women taking into account the impact of antiretroviral therapy.

Early age at first intercourse was associated with positive *T. vaginalis* infection in our study. Similar results have been reported by other studies such as in China among 734 FSW (aOR = 1.9 [95% CI: 1.11–3.30]) for starting age in commercial sex before 20 years [31] and in India in 2006 (aOR = 2.09; 95%CI: 1.09–4.00) [32]. Overall prevalence of *T. vaginalis* among sexually active women aged 15–30 years was 8.5 and 14.4% for women under 50 years at first sex. Another study of a nationally representative sample of 9844 respondents aged 18 to 26 years in the United States found a significantly higher risk of *T. vaginalis* infection among adolescents and young adults who were younger at the time of their first sexual intercourse [33]. Also consistent with our result, a cross-sectional study conducted in four cities in sub–Saharan Africa (Kisumu, Kenya; Ndola, Zambia; Cotonou, Benin and Yaoundé, Cameroon) among a random sample of 8000 adults (2000 in each city), aged 15–49 years showed a prevalence of *T. vaginalis* respectively of 29.3% in Kisumu, 34.3% in Ndola, 3.2% in Cotonou and 17.6% in Yaoundé. Early sexual debut (before age 15) was a significantly risk factor associated with *T. vaginalis* infection in women in Ndola (Zambia) [34]. Specific interventions are needed to delay the age of the first sexual intercourse among FSW and in general population.

Most of studies on *T. vaginalis* in Africa are conducted in pregnant women and report high prevalence in this population. A nested case-control study in Kenya among pregnant women reported a *T. vaginalis* infection prevalence of 35.4% (*n* = 79) [35], while in Nigeria [36] and South Africa [37], the prevalence of *T. vaginalis* infection among pregnant women was 18.7 and 15.0%, respectively. Hence, in many studies in sub-Saharan Africa, the prevalence of *T. vaginalis* among women in the general population, especially among pregnant women is higher than that of FSW, as reported in this study. One plausible explanation could be the use of molecular biology test, which is among the most specific for *T. vaginalis* detection compared to other tests that could be less accurate. Another possible explanation could be the systematic use of treatment in case of genital infection for FSW. In Togo, in case of STI symptoms, syndromic approach which includes the use of azithromycin, ceftriaxone, doxycycline, metronidazole as first line treatment are systematically used in care centers. However, additional and comparative studies are needed to shed light on interventions or hypotheses that could explain it.

To our knowledge, this was the first study reporting prevalence of *T. vaginalis* infection and other STI among FSW in Togo. Another strength of this study includes the use of a sensitive laboratory assay for the reliable detection of *T. vaginalis* infection and the relationship with HIV. Finally, our study was the first to assess factors associated with *T. vaginalis* among FSW in Togo and to provide useful information in order to design specific interventions within these populations.

There were few limitations to this study including the lack of data on treatment use among study participants, which may have certainly impacted observed STI prevalence. In Togo, syndromic approach is used for the treatment of STI among FSW and in general population. Furthermore, the standardized questionnaire submitted to participants can be biased (memory bias and social desirability bias) by the fact that it was based on self-reporting and may not reflect the overall sexual activity. Additionally, due to the cross-sectional nature of this analysis, we are unable to analyze the causality and temporality of the associations between *T. vaginalis* infection and other factors.

## Conclusion

The prevalence of *T. vaginalis* infection using molecular test among FSW in Togo was low. However, extensive studies are needed to confirm and better understand the epidemiology of *T. vaginalis* in these populations in Togo. Comprehensive health promotion programs for FSW and active surveillance that include preventive education are needed.

## Data Availability

The datasets used and/or analysed during the current study are available from the corresponding author on reasonable request.

## Abbreviations

95%CI: 95% Confidence interval
aOR: Adjusted odds ratio
*T. vaginalis*: Trichomonas vaginalis
